# Assessment of *T*_1_ and *T*_2_ relaxation times of deuterium (^2^H) labeled resonances in the human liver and kidney using *k*-space reordered 3D concentric ring trajectory sampling at 7T

**DOI:** 10.1007/s10334-025-01320-9

**Published:** 2026-01-16

**Authors:** Viola Bader, Bernhard Strasser, Lukas Hingerl, Johannes J. Kovarik, Sabina Frese, Lorenz Pfleger, Anna Duguid, Aaron Osburg, Martin Krššák, Thomas Scherer, Wolfgang Bogner, Fabian Niess

**Affiliations:** 1 High-Field MR Center, Department of Biomedical Imaging and Image-Guided Therapy, Medical University of Vienna, Vienna, Austria; 2 Clinical Division of Internal Medicine III, Department of Nephrology and Dialysis, Medical University of Vienna, Vienna, Austria; 3 Clinical Division of Internal Medicine III, Department of Endocrinology and Metabolism, Medical University of Vienna, Vienna, Austria; 4 Christian Doppler Laboratory for MR Imaging Biomarkers (BIOMAK), Department of Biomedical Imaging and Image-Guided Therapy, Medical University of Vienna, Vienna, Austria; 5 Comprehensive Center of Artificial Intelligence in Medicine (CAIM), Medical University of Vienna, Vienna, Austria

**Keywords:** Deuterium metabolic imaging, DMI, Relaxation time measurements, 3D magnetic resonance imaging, Glucose metabolism

## Abstract

**Objective:**

Deuterium metabolic imaging (DMI) is an emerging MR technique providing non-invasive insights into glucose metabolism. Reliable concentration estimation depends on knowledge of tissue specific relaxation times. This study reports *T*₁ and *T*₂ relaxation time constants of deuterium-labeled water (HDO) and glucose (Glc) from the human liver and kidney at 7T.

**Materials and methods:**

Twelve healthy volunteers (6f/6 m) were examined using *k*-space-reordered inversion-recovery and spin-echo DMI with non-Cartesian concentric-ring trajectory (CRT) sampling. Seven volunteers underwent oral ^2^H-Glc (0.8 g/kg body weight) administration. Data were averaged over organ-specific masks before spectral fitting. One volunteer was measured after oral D₂O (0.5 ml/kg body weight) administration.

**Results:**

Faster longitudinal relaxation but similar transversal relaxation were observed for ^2^H-labeled Glc in the liver compared to kidney tissue (*T*₁^liver/kidney^ = 60 ± 4 ms/85 ± 18 ms, *p* = 0.016; *T*₂^liver/kidney^ = 31 ± 6 ms/35 ± 2 ms, *p* = 0.283). HDO exhibited significantly shorter liver relaxation times (*T*_1_^liver/kidney^ = 218 ± 24 ms/324 ± 34 ms, *p* < 0.001; *T*₂^liver/kidney^ = 28 ± 4 ms/39 ± 6 ms, *p* < 0.001). D₂O loading improved voxelwise SNR enabling renal *T*₁/*T*₂ mapping of HDO.

**Discussion:**

Hepatic and renal glucose homeostasis is often impaired in several pathophysiological conditions such as tumors, diabetes and metabolic dysfunction-associated steatotic liver disease. Using organ-specific ^2^H relaxation times increases the accuracy of concentration estimation and can help to improve the understanding of underlying metabolic processes in future abdominal DMI studies, which can help to push abdominal DMI towards clinical application.

**Supplementary Information:**

The online version contains supplementary material available at 10.1007/s10334-025-01320-9.

## Introduction

Magnetic resonance spectroscopic imaging (MRSI) of deuterium-labeled metabolites has recently emerged as a promising approach to non-invasively monitor metabolic processes in vivo [1, 2]. Following oral or intravenous administration of deuterated substrates, e.g. deuterium (^2^H)-labelled glucose (Glc), deuterium metabolic imaging (DMI) allows dynamic mapping of ^2^H-Glc uptake and downstream metabolism. Given the central role of altered Glc metabolism in many diseases, e.g., tumors, neurodegenerative diseases, diabetes, metabolic dysfunction-associated steatotic liver disease [3–8], DMI offers valuable complementary information to conventional imaging by providing direct insight into biochemical processes.

Most in vivo DMI applications to date have focused on the brain [1, 2, 9–11]. Extending DMI beyond the brain is of high clinical interest as, e.g., the liver and kidney play a central role in whole body glucose homeostasis [8, 12]. The liver coordinates systemic gluconeogenesis, glycogen storage, release and redistribution [8, 13], whereas the kidney coordinates salt and water homeostasis and glucose reabsorption [14, 15]. Establishing robust abdominal DMI could therefore deepen the clinical understanding of hepatic and renal physiology and pathophysiology, which is often linked to abnormal glucose metabolism [6, 7].

Translating DMI to the abdomen has only recently been demonstrated [16–23], but remains challenging due to lower signal-to-noise ratio (SNR), spectral quality and depth-dependent *B*_1_^⁺^ inhomogeneity attributed to the use of surface coils. Increased tissue inhomogeneity, motion, suboptimal *B*_0_ shimming, and spatial-resolution constraints when using conventional phase-encoding readouts introduce further impediments. First abdominal studies proved feasibility of DMI at clinical 3T [16, 17] and higher field strengths of 7T [18–22], providing first dynamic time courses of Glc uptake after oral tracer administration of ^2^H-labelled Glc. Recently, feasibility of dynamic renal DMI at 7T using fast concentric-ring readouts with sub-milliliter nominal voxel volumes (~ 0.9 mL) was reported by our research group [24], indicating the potential of abdominal DMI for clinical application.

A critical missing piece for quantitative abdominal DMI studies are organ-specific relaxation times, as accurate metabolite concentration estimation requires knowledge of tissue-specific relaxation times [25]. Simple unlocalized FID-based relaxation time measurements, as often performed in the brain [1, 10, 26], are not practical for tissues located deep in the human body (i.e., liver or kidney), because acquired data would yield a composite signal from multiple organs. However, minimum achievable slice thickness when using single-voxel localization approaches is limited by strong gradient demands due to the low gyromagnetic ratio of ^2^H. Furthermore, MRSI sequences using conventional 2D or 3D phase encoding often feature prolonged scanning time for higher matrix sizes and therefore, are not feasible for time-sensitive tracer experiments with varying concentrations over time. Consequently, liver- and kidney-specific literature values remain sparse.

We have previously proposed an inversion-recovery/Hahn spin-echo 3D ^2^H-MRSI sequence [27], which combines rapid spatial-spectral encoding using Hamming-weighted concentric ring trajectory (CRT) readout with specific *k*-space reordering of the ring trajectories. This novel sampling scheme improves robustness to time-varying metabolite concentrations typically present in tracer experiments. The sequence was previously validated in the brain delivering tissue-specific WM and GM dominated relaxation times but is highly versatile and can be adapted for use in other organs. As inherently low SNR is a common problem in DMI, relaxation time fitting was performed after averaging over regions of interest using organ specific masks derived from high resolution ^1^H MRI images, whereas D_2_O dosing additionally allowed voxelwise fitting.

In this study, we demonstrate abdominal implementation of a previously developed sequence to assess tissue-specific *T*_1_ and *T*_2_ relaxation times for natural abundant ^2^H-water and ^2^H-Glc after oral tracer administration in the human liver and kidney.

## Materials and methods

### Study participants

Twelve healthy subjects (6f/6 m, age 29 ± 5, BMI 23 ± 2 kg/m^2^) without a medical history of metabolic diseases were enrolled in this study after written informed consent was obtained. The study was approved by the local ethics committee of the Medical University of Vienna according to the guidelines of the Declaration of Helsinki. Sequence parameters were optimized in phantom and in vivo using the natural abundance ^2^H-water signal to ensure robust SNR and stable fitting.

Natural abundance scans (no tracer) were acquired in six volunteers to measure ^2^H-water relaxation times in the kidney or liver; one volunteer was scanned twice, targeting different organs in separate sessions. For abdominal relaxation-time measurements of ^2^H-Glc, participants were scanned in an overnight fasted state in the morning after oral administration of ^2^H-labeled Glc (0.8 g/kg body weight, [6,6’]-^2^H-Glc ≥ 99% purity, Cambridge Isotopes) dissolved in ~ 200 ml water. Seven participants underwent Glc administration of which two were rescanned after having completed natural abundance sessions on a separate day. To facilitate renal relaxation time mapping of ^2^H-water, one subject consumed heavy water (D₂O, 0.5 ml/kg body weight in ~ 200 ml drinking water) the day prior to the measurement to substantially increase the SNR, which enabled voxelwise relaxation time fitting with higher spatial resolution. An overview of subject inclusion per analysis is provided in Tables 1 and 2 for ^2^H-Glc and ^2^H-water, respectively.

**Table 1:** *T*_1_ (a, left) and *T*_2_ (b, right) relaxation times [ms] of ^2^H-Glc in the human kidney and liver which were acquired after oral administration of ^2^H-labeld Glc

a	*T*_1_ [ms]	
Subject	kidney	liver
3		60 ± 8
6	103 ± 14	67 ± 5
7		55 ± 12
8	104 ± 24	58 ± 4
9	63 ± 8	
10	83 ± 12	59 ± 6
11	74 ± 14	
Mean ± SD	85 ± 18*	60 ± 4

b	*T*_2_ [ms]	
Subject	kidney	liver
3		37 ± 4
6	35 ± 10	
7	36 ± 5	25 ± 3
8		38 ± 8
9	31 ± 2	31 ± 1
10	37 ± 4	
11	35 ± 4	27 ± 2
Mean ± SD	35 ± 2	31 ± 6

Data was acquired using 3D CRT-based relaxation time MRSI. Statistical difference (*) was found between *T*_1_ kidney and liver relaxation times

**Table 2:** *T*_1_ (a, left) and *T*_2_ (b, right) relaxation times [ms] of ^2^H-water in the human kidney and liver which were acquired in 12 healthy volunteers without tracer administration

a	*T*_1_ [ms]	
Subject	Kidney	Liver
1	329 ± 22	234 ± 1
2		250 ± 16
3	305 ± 49	
4		213 ± 6
5	326 ± 49	195 ± 12
6	289 ± 31	196 ± 11
12	371 ± 51	
Mean ± SD	324 ± 31*	218 ± 24

b	*T*_2_ [ms]	
Subject	Kidney	Liver
1	42 ± 7	
2	32 ± 4	33 ± 4
3	36 ± 4	30 ± 2
4		23 ± 2
5	32 ± 7	27 ± 2
6	52 ± 5	
7	34 ± 3	24 ± 2
8		33 ± 5
9	42 ± 5	30 ± 1
10	42 ± 5	
11	42 ± 5	27 ± 2
12	39 ± 3	
Mean ± SD	39 ± 6*	28 ± 4

Data was acquired using 3D CRT-based relaxation time MRSI. Statistical differences (*) was found for both *T*_1_ and *T*_2_ relaxation times between the two organs. Subject 12 was measured with higher resolution (32 × 32 × 31) after D_2_O loading the day prior to the measurement

### Measurement protocol

#### General setup

**Fig. 1:**
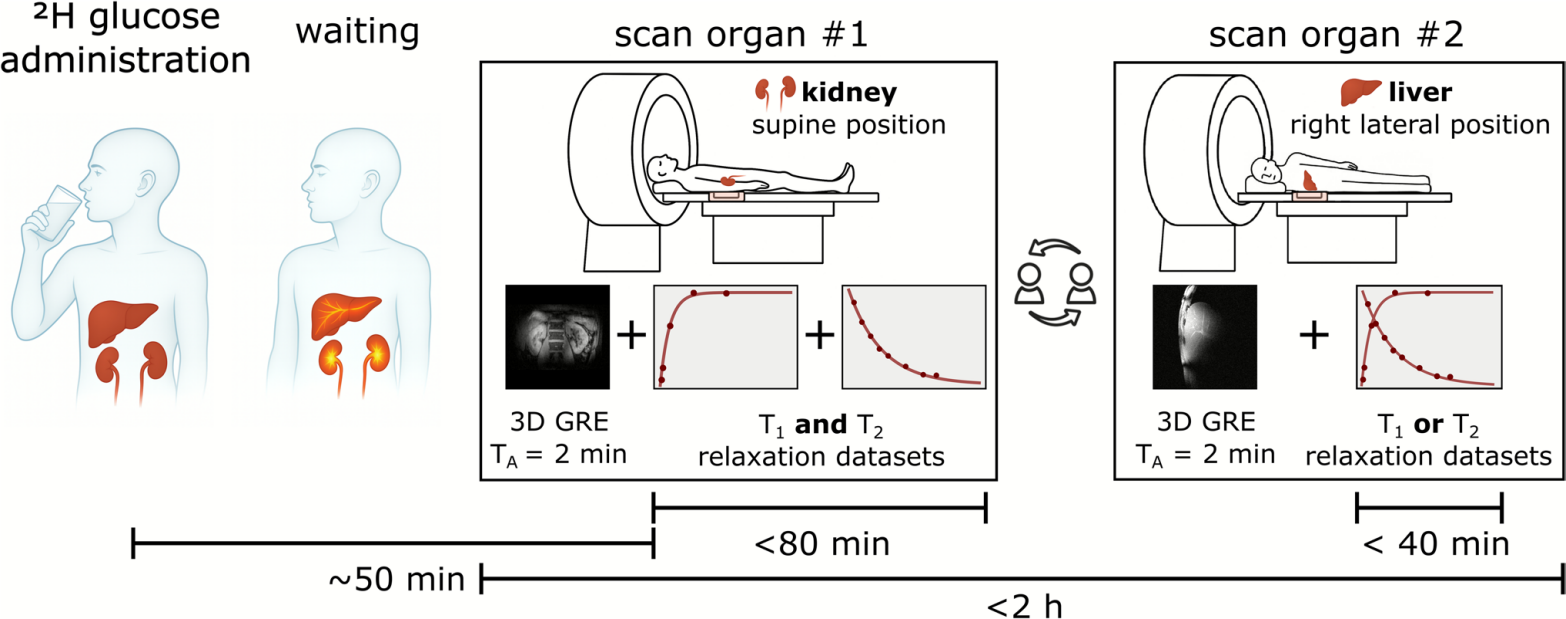
Overview of the ^2^H-Glc relaxation time protocol. After oral ^2^H-Glc administration, participants were positioned in the scanner ~ 40 min post-dose; 3D CRT-based ^2^H-MRSI relaxation measurements began ~ 50 min after administration. Both organs were examined within the same session; organ order and *T*₁/*T*₂ acquisition order were alternated across subjects to avoid bias (the protocol shown here is illustrative). In each organ 3D GRE and multi-slice localizer anatomical images were acquired followed by acquisition of two (first organ) or one (second organ) T₁/T₂ datasets. Participants were removed from the bore between organs scans for repositioning and setup change. Total session time averaged < 2 h. Natural abundance scans were performed without tracer administration and usually only *T*_*1*_ and *T*_*2*_ datasets in one organ were acquired, scan of the second organ was only performed in two volunteers

All measurements were performed on a 7T whole-body MR scanner (Magnetom dot Plus, Siemens, Healthineers, Erlangen, Germany) using a dual-tuned (^2^H/^1^H) body coil array [one ^2^H transmit channel (~ 27 × 27 cm), two ^2^H receive channels: one loop (~ 17 × 17 cm) and one butterfly (~ 25 × 15 cm)], Stark Contrasts MRI Coils Research, Germany). Subject positioning was adapted to the target organ: for kidney measurements, participants were positioned supine with the dual-tuned ^2^H/^1^H surface coil array placed posterior to the lower back in a foamed-plastic insert, similar to the setup described previously [24]. For liver measurements, subjects were positioned in right lateral position, with the coil placed beneath the lower right rib cage, stabilized with sandbags to improve comfort, see Fig. 1.

#### MR(S)I protocol

Prior to relaxation time measurements, protocol included tri-planar localizer images, which were used to verify coil placement and plan the spectroscopic volume followed by 3D gradient-echo anatomical imaging (FOV 340 × 340 × 224 mm^3^; matrix 256 × 256 × 160; *TR* = 3.4 ms; *TE* = 1.38 ms; *TA* = 1:55 min). Shimming was performed using the vendor-supplied 3D shimming routine (“DESS” followed by “GRE-BREAST”) with the shim volumed adjusted to cover the target organ (either both kidneys or the liver).

For this study, a previously developed 3D ^2^H-FID-MRSI sequence was used, which combines fast Hamming-weighted non-Cartesian concentric ring trajectory (CRT) sampling with integrated *k*-space reordering [27], enabling spatially resolved measurements of the relaxation times (*T*_1_ or *T*_2_) of ^2^H-labeled resonances, i.e., ^2^H-water, ^2^H-Glc. Robustness against concentration changes was achieved by reordering the ring trajectories in a way that each ring was sampled several times with increasing *TI*/*TE* before moving to the next larger ring, ensuring that different *TI*/*TE* times for each *k*-space point are sampled in a short period of time. This, in combination with sampling the *k*-space center outwards, makes the sequence robust against concentration changes of metabolic compounds, which was extensively tested on simulated datasets in a prior study [27]. For abdominal applications (kidney and liver), the sequence was adapted and optimized to comply with specific absorption rate (SAR) constraints associated with surface coils due to high required reference voltages and to maintain feasible examination times: RF pulse durations were increased, and the nominal spatial resolution was reduced relative to the original brain implementation due to lower abdominal SNR.

Transmit calibration and flip-angle selection were based on prior calibration performed on a torso phantom using double-angle *B*₁⁺ mapping [24] showing that a reference voltage *U*_ref_ of 400 V yields ~ 45° flip angle, implying that 800 V reference voltage is required to achieve a 90° flip angle, which was also supported by in vivo kidney scans showing comparable spectral SNR for 720-840 V. Thus, for all relaxation time measurements, a reference of 800 V for 90° flip angle was chosen. Because incomplete inversion was observed at renal depth, the voltage of the inversion pulse for kidney *T*₁ experiments was increased by 25%. For the liver, the reference voltage was left unchanged owing to the shorter coil-to-organ distance in right-lateral positioning. Final settings were: *T*₁^kidney^: inversion pulse 4 ms and excitation pulse 2 ms; *T*₁^liver^: inversion pulse 3 ms and excitation pulse 2 ms; *T*₂^both organs^: excitation pulse 3 ms and refocusing pulse 3.5 ms, all pulses used were of rectangular shape. All parameters were verified in phantom to check if 90° and 180° flip angles were reached and the repetition time was adjusted to stay withing SAR limits.

#### Natural abundance scans

Following anatomical ^1^H imaging, *T*₁ and *T*₂ relaxation-time datasets were typically acquired for a single organ within one session. In two volunteers, an additional *T*₁ or *T*₂ measurement in the second organ was appended after repositioning, see Fig. 1. MRSI acquisition parameters were: FOV 270 × 270 × 260 mm^3^; matrix 18 × 18 × 17; nominal isotropic voxel 3.44 ml; *N*_circles_ = 21; readout bandwidth 380 Hz; spectral points 96; *TR* = 900-1150 ms; *T*_I_s: 5, 50, 450, 650, 900 ms; *T*_E_s (n = 6): 8, 10, 15, 20, 40, 60 ms; *TA* = 28-42 min; acquisition delay = 2.0 ms (*T*₁)/0 ms (*T*₂). *TR* was adjusted on a per-subject basis to remain within SAR limits, depending on coil loading and individual positioning.

#### ^2^H-Glc scans

Given the higher cost of ^2^H-Glc measurements, the protocol was extended to minimize costs: after acquisition of *T*₁/*T*₂-datasets in the first organ, an additional dataset (*T*₁ or *T*₂) in the second organ was obtained.

After ingestion, participants were positioned on the patient bed and moved inside the scanner bore ~ 40 min post ^2^H-Glc administration. Following localizers, anatomical imaging and shimming, 3D CRT ^2^H-MRSI relaxation measurements started ~ 50 min after tracer consumption. For one target organ, two datasets (*T*₁ and *T*₂) were acquired consecutively and for the other organ one additional dataset (*T*₁ or *T*₂) was acquired. Participants were removed from the scanner bore in between the two organ scans and repositioned after coil/setup adjustments. Acquisition order (*T*₁/*T*₂ and organ) was alternated across subjects to avoid bias. *TR*s were adjusted for each subject to remain within SAR limits. Overall measurement time was under 2 h. The experimental protocol and timing are summarized in Fig. 1.

MRSI parameters matched the natural abundance protocol, with the exception that *TI* spacing was optimized for faster glucose relaxation (*TI* = 5, 15, 70, 150, 500 ms). Echo times (*TE*) for scans including ^2^H-Glc tracer administration were identical to the natural abundance *T*₂ protocol; consequently, *T*₂ times of ^2^H-water and ^2^H-Glc could be derived from the *T*_2_ tracer datasets.

#### High-resolution D_2_O scans

One subject was scanned after oral D₂O ingestion the day prior to the measurement. Acquisitions were performed at an isotropic resolution of 0.6 ml (matrix: 32 × 32 × 31, acquisition delay = 2.5 ms (*T*₁)/0 ms (*T*₂), *TA*^T1/T2^ = 40/46 min) with otherwise similar acquisition parameters as described above for natural abundance scans. Due to prolonged scan time *k*-space was sampled equidistant instead of Hamming-weighted followed by a 3D Hamming-Filter during post-processing.

For detailed information about sequence parameters see Table S1 for minimum reporting standards [28].

### Data postprocessing

Data reconstruction was performed using in-house developed post-processing (MATLAB R2021, LCModel v6.3, Python v3.10) including non-Cartesian three-dimensional discrete Fourier transformation without density compensation [11, 29, 30]. Channels were combined using Whitened Singular Value Decomposition (WSVD) [31, 32] followed by spectral denoising with tensor Marchenko-Pastur PCA (tMPPCA) (datashape [4 5 1:2 3], patch size: matrix dimension/4) to stabilize spectral fitting of low SNR data [33, 34].

Analogous to our previous study in the brain [27], we report only region specific relaxation times, expect for one dataset obtained after D_2_O dosing. Therefore, spectra were averaged within organ specific masks, while preserving spatial specificity. Kidneys and liver were manually segmented on high-resolution ^1^H 3D GRE images in ITK-SNAP [35], masks were downsampled to MRSI grid using MINC tools (MINC tools, v2.0, McConnell Brain Imaging Center, Montreal, QC, Canada) and only voxels with LCModel SNR > 3 were included in the analysis. This criterion was used solely to remove voxels with clearly insufficient spectral quality and should not be interpreted as an absolute or universally applicable SNR threshold. Before averaging, voxelwise *B*₀ correction was performed using LCModel-derived frequency shift maps to realign spectra, followed by zero-order phasing to account for spatially different phase and frequency followed by spectral fitting using LCModel. High-resolution kidney datasets acquired after D₂O loading provided sufficiently high SNR for voxelwise *T*₁/*T*₂ mapping. Voxels exhibiting high exponential fitting error (SD > 50%) were excluded from the analysis.

For spectral fitting, a custom build basis set was generated containing ^2^H-water (4.8 ppm) and ^2^H-Glc (3.9 ppm), with acquisition delay settings chosen to match first-order phase (*T*₁^low−/high−resolution^: 2/2.5 ms; *T*₂: 0 ms). Because ^2^H-water and ^2^H-Glc exhibit markedly different *T*₁ values, the basis set unconventionally included corresponding phase-inverted (180°) components. The *TI*-appropriate basis was selected automatically during fitting, based on estimated relaxation times of the metabolites, i.e. for short and long *TI* values both peaks are in phase, whereas at intermediate *TI* the glucose signal has already recovered to positive phase while the water signal remains inverted. Natural abundance scans were modelled with a ^2^H-water-only basis set.

### Relaxation time fitting and statistical analysis

Exponential fitting of relaxation times was performed using the following equations: $M\left({T}_{I}\right)={C}_{1}(1-{C}_{2}*{e}^{-{T}_{I}/{T}_{1}})$ and $M\left({T}_{E}\right)={C}_{1}*{e}^{-{T}_{E}/{T}_{2}}$, assuming a 3 and 2 parameter fit for *T*_1_ and *T*_2_ experiments, respectively. To asses significant differences between the two tissues paired *t*-test was applied, due to small sample sizes [36], with a statistical significance threshold of *p* < 0.05. Relaxation time exponential fitting and statistical analysis were performed using python (v3.10, curve_fit function from scipy.optimize and scipy.stats).

SNR was calculated for averaged spectra for the first *TI*/*TE* using the amplitudes of ^2^H natural abundant water and the standard deviation of the noise in a region ∼100 Hz off-resonance. For measurements performed after ^2^H-Glc administration, SNR was additionally calculated for the ^2^H-Glc peak using the same noise definition. For high-resolution kidney dataset SNR was calculated voxelwise and reported as the mean over both kidneys. Full Width at Half Max (FWHM), and Cramer-Rao Lower Bounds (CRLBs) were estimated using LCModel. It should be noted that denoising alters noise characteristics, therefore SNR as well as CRLB values derived from denoised data should not be interpreted as absolute or generally comparable quality metrics. In this study, they are reported only as relative indicators of spectral quality within our datasets.

## Results

**Fig. 2:**
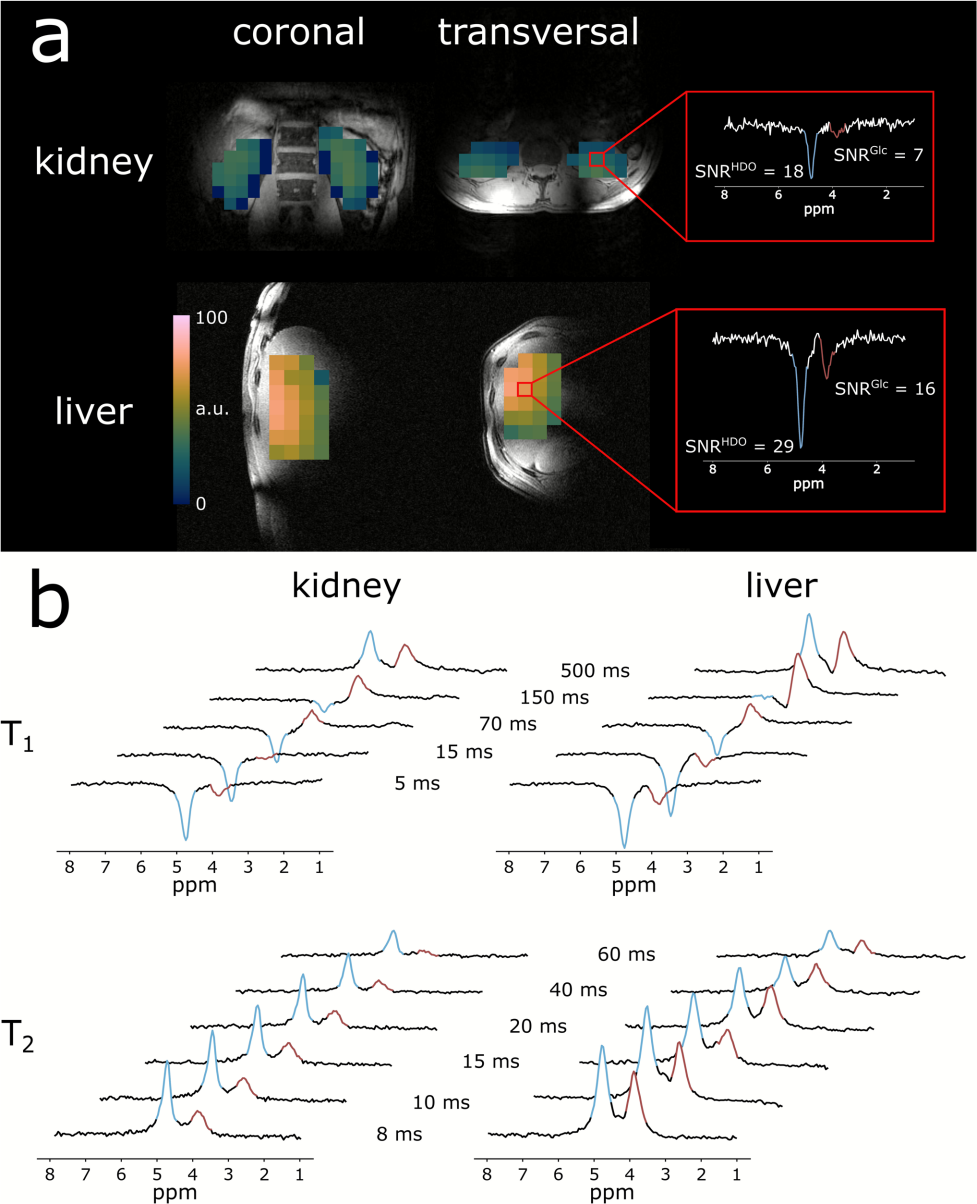
**a** Coronal and transverse anatomical images of kidney (top) and liver (bottom) with overlaid ^2^H-water amplitude maps from the first *TI* = 5 ms. Identical scaling highlights the higher SNR in the liver. Outlines indicate the organ-specific masks used for spectral averaging. Spectral signal (first *TI*) of two exemplary voxels before spectral averaging is shown. **b** Representative high SNR spectra averaged within organ masks across increasing inversion times (T₁, top) and echo times (T₂, bottom) for kidney (left) and liver (right). Data were acquired after oral ^2^H-Glc administration; spectra therefore show ^2^H resonances from natural abundance ^2^H-water (blue) and ^2^H-Glc (red)

Organ-specific masks were manually segmented. As some scans acquired after tracer administration exhibited strong contaminating signal from stomach or smaller intestines, masks were conservatively segmented excluding voxels with unreasonably high glucose signal (threshold: ^2^H-Glc / ^2^H-water ratio > 1.5). Additionally, all masks were inspected visually, voxels with unphysiologically high signal were conservatively excluded from the analysis. Averaging within these masks substantially increased SNR (SNR of water peak for first *TI*/*TE*: inversion recovery: SNR^kidney^ = 55 ± 22, SNR^liver^ = 82 ± 15; spin-echo: SNR^kidney^ = 55 ± 29, SNR^liver^ = 90 ± 30). For measurements performed after ^2^H-Glc administration, the corresponding averaged ^2^H-Glc SNR also increased substantially (SNR of ^2^H-Glc peak for first *TI*/*TE*: inversion recovery: SNR^kidney^ = 14 ± 5, SNR^liver^ = 43 ± 12; spin-echo: SNR^kidney^ = 22 ± 3, SNR^liver^ = 43 ± 16). Figure 2a illustrates representative water amplitude maps for kidney and liver and shows low-SNR spectra from two representative voxels.

### ^2^H-Glc

In vivo relaxation time measurements started 52 ± 6 min after oral ^2^H-Glc administration. The second MRSI scan started after 96 ± 19 min, followed by the final scan 151 ± 16min post dosage. Repositioning was performed either after the first or the second scan and order of *T*_1_/*T*_2_ and starting organ was randomized to avoid bias. Relaxation time measurements took on average 35 ± 3min per scan, depending on the *TR* which had to be adjusted on a volunteer basis to remain within the SAR limits. Representative averaged high-SNR spectra series illustrating the signal evolution for increasing *TI*/*TE* are shown in Fig. 2b. The CRLBs of averaged spectra were below 10% and 26% for liver and kidney, respectively. SNR, FWHM, number of averaged voxels and ^2^H-Glc/^2^H-water ratios of the first *TI*/*TE* are listed in Table S2.

**Fig. 3:**
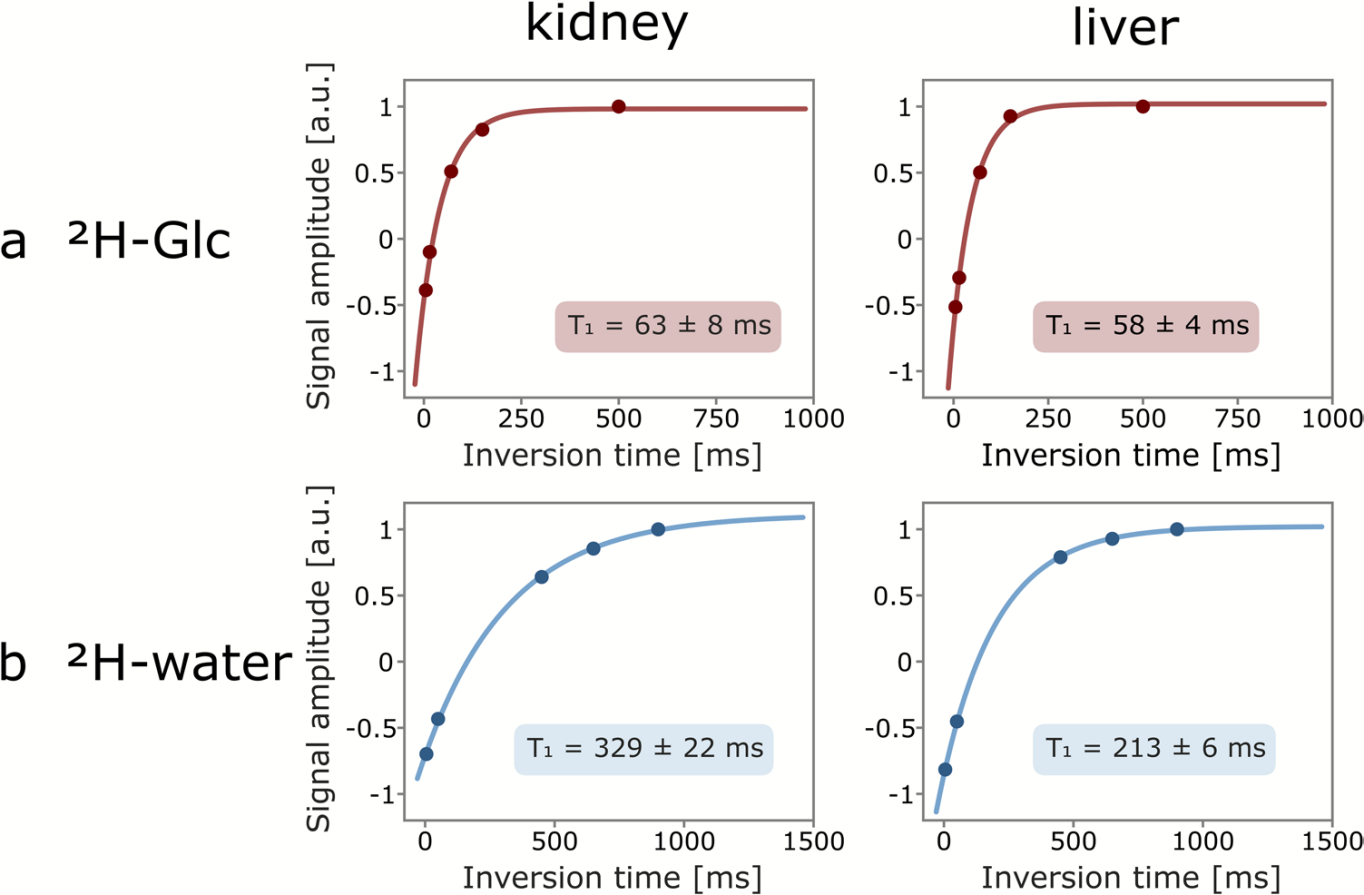
Exemplary exponential fits to estimate abdominal *T*_1_ relaxation constants of ^2^H-Glc (a, red) and ^2^H-water (b, blue) from two healthy volunteers. Scans were performed after oral administration of ^2^H-labeled Glc (**a**) and without tracer administration (**b**) using a 3D CRT based ^2^H-MRSI sequence with specific reordering of the *k*-space, which allowed to regionally detect relaxation times in the kidney (left) and liver (right)

**Fig. 4:**
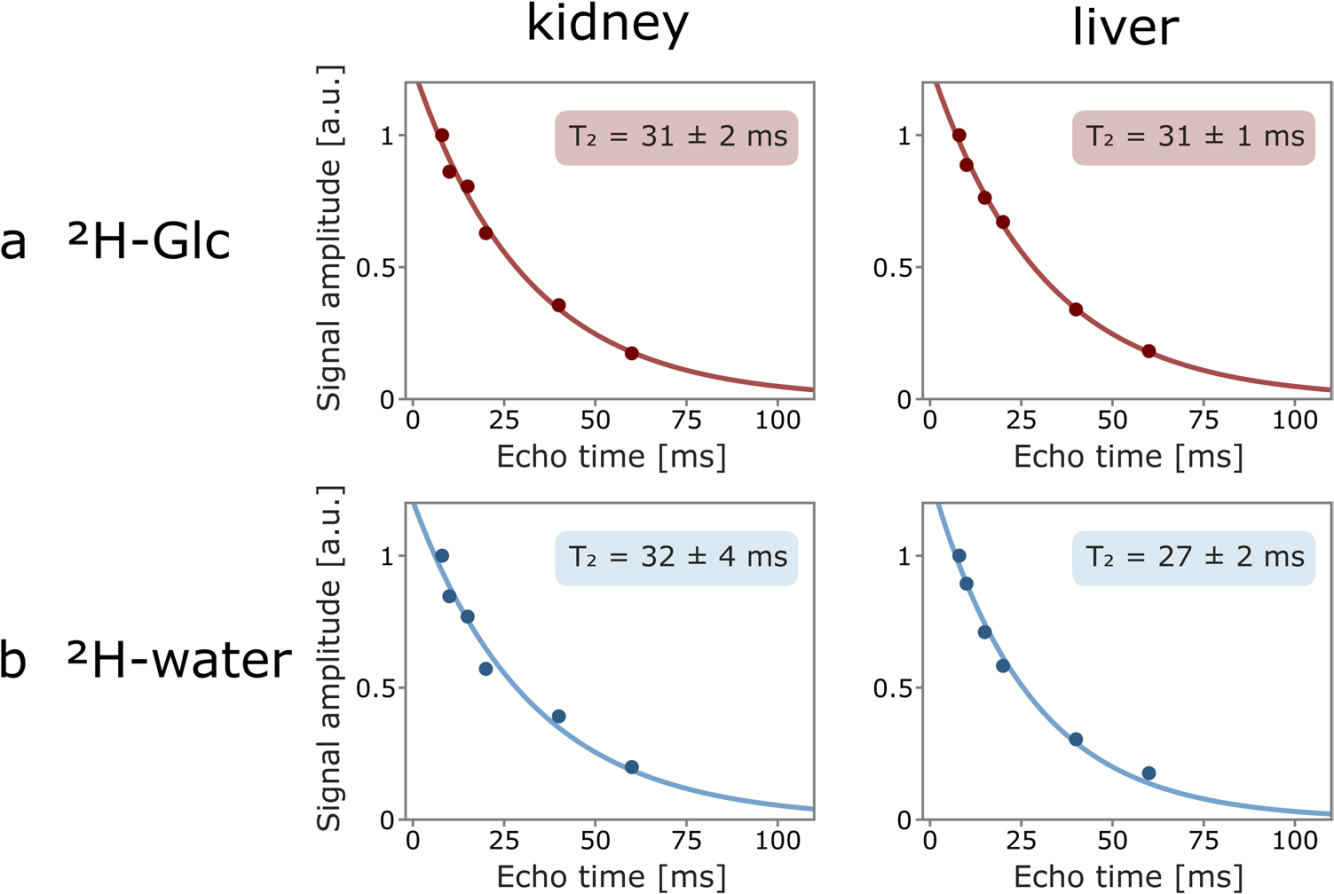
Exemplary exponential fits to estimate abdominal T_2_ relaxation constants of ^2^H-Glc (a, red) and ^2^H-water (b, blue) from two healthy volunteers. Scans were performed after oral administration of ^2^H-labeled Glc (**a**) and without tracer administration (**b**) using a 3D CRT based ^2^H-MRSI sequence with specific reordering of the *k*-space, which allowed to regionally detect relaxation times in the kidney (left) and liver (right)

Significantly longer *T*_1_ relaxation times were observed in the kidney compared to the liver (*T*_1_^Glc_liver^ = 60 ± 4 ms, *T*_1_^Glc_kidney^ = 85 ± 18 ms, *p* = 0.016), whereas similar *T*_2_ relaxation times were assessed (*T*_2_^Glc_liver^ = 31 ± 6 ms, *T*_2_^Glc_kidney^ = 35 ± 2 ms, *p* = 0.283). *T*_1_ relaxation estimation in the kidney featured higher inter-subject variability than in the liver. Individual results of all volunteers are summarized in Table 1 and representative exponential fits of one volunteer are shown in Fig. 3a and Fig. 4a.

### Natural abundance ^2^H water

Relaxation times of natural abundant ^2^H-water resonances were acquired without tracer administration and were therefore not time sensitive, hence typically one organ (*T*_1_ and *T*_2_) was measured per session. Averaged high SNR spectra featured CRLBs below 13%; SNR, FWHM and number of averaged voxels are listed in Table S2. Significantly shorter relaxation time constants were found in the liver (*T*_1_^water_liver^ = 218 ± 24 ms, *T*_1_^water_kidney^ = 324 ± 31 ms, *p* < 0.001) (*T*_2_^water_liver^ = 28 ± 4 ms, *T*_2_^water_kidney^ = 39 ± 6 ms, *p* < 0.001). Individual results of all volunteers are summarized in Table 2 and representative exponential fits of one volunteer are shown in Fig. 3b and Fig. 4b.

### High-resolution relaxation time mapping in the kidney

Oral administration of D_2_O water substantially increased the renal ^2^H-labeled water concentration and allowed to increase the nominal spatial resolution by a factor of 5.7 from 3.44 to 0.6 ml, ultimately enabling *T*_1_ and *T*_2_ mapping of the kidney. Voxelwise SNR was comparable between natural abundance scans with lower spatial resolution (SNR^T1/T2^ = 14 ± 3/14 ± 5) and scans after D_2_O administration with higher spatial resolution (SNR^T1/T2^ = 17 ± 6/19 ± 9).

**Fig. 5:**
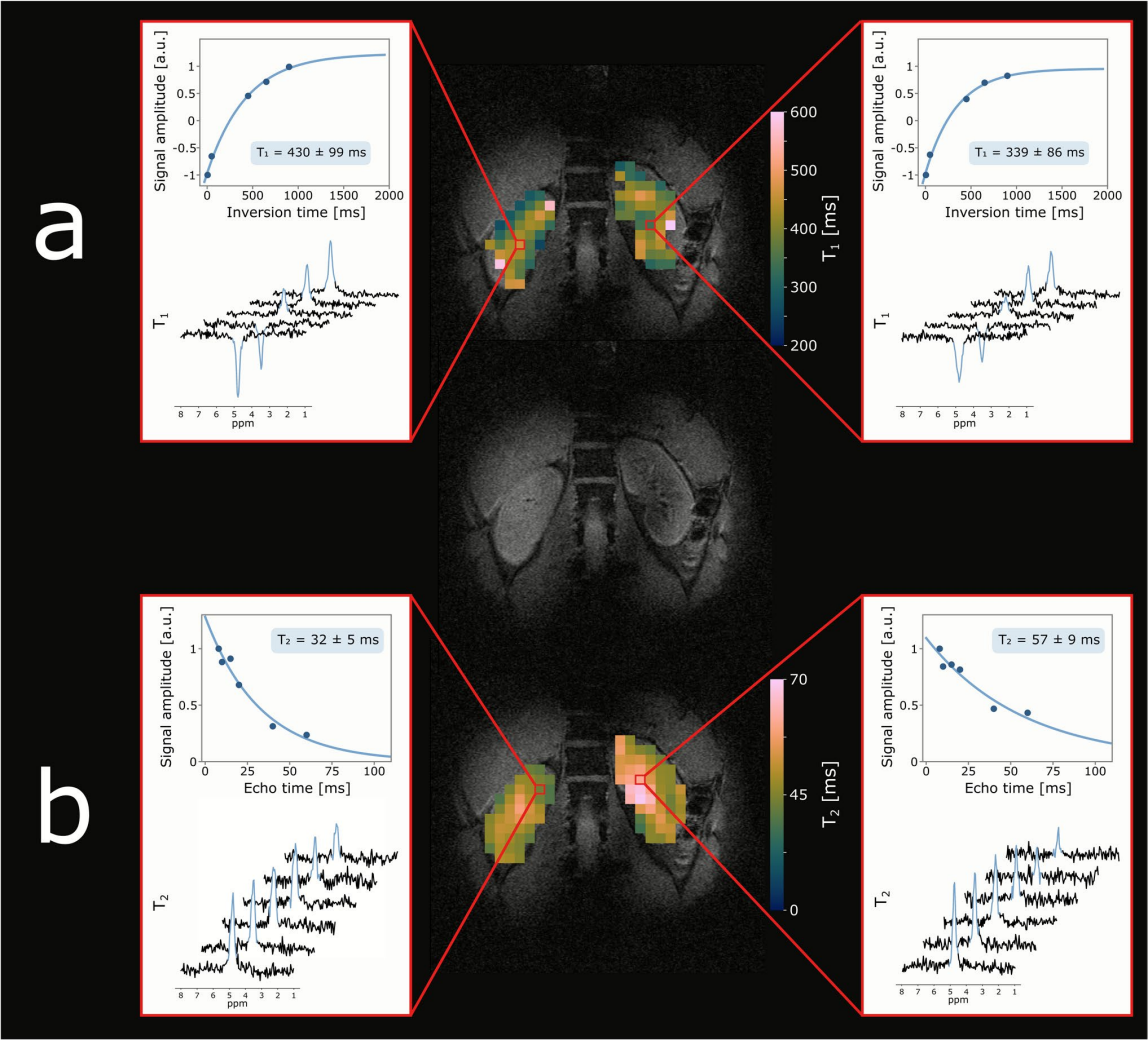
Coronal *T*_1_ (**a**) and *T*_2_ (**b**) relaxation time maps acquired at increased special resolution (32 × 32 × 31) of one healthy volunteer, who was measured after consuming D_2_O the day prior to the measurement. Exponential fits and evolution of sample spectra for increasing *TI*/*TE* are exemplarily shown for two voxels

Coronal *T*_1_ and *T*_2_ maps of the kidney, as well as representative sample spectra and fits of two exemplary voxels are shown in Fig. 5. Comparing the mean of all voxelwise fitted relaxation time constants versus averaged (before spectral fitting) high SNR relaxation constants yielded similar results: *T*_1_^voxelwise^ = 404 ± 90 ms, *T*_1_^averaged^ = 371 ± 51 ms and *T*_2_^voxelwise^ = 43 ± 9 ms, *T*_2_^averaged^ = 39 ± 3 ms.

## Discussion

This study demonstrates the feasibility of measuring organ-specific relaxation times of ^2^H-water and ^2^H-Glc in the human liver and kidney using a previously described 3D ^2^H-MRSI sequence with Hamming-weighted non-Cartesian CRT readout [27]. The sequence includes specific *k*-space reordering of the ring trajectories, enabling robust relaxation time estimation even when metabolite concentrations vary during acquisition. This is especially important for in vivo measurements where a steady state over a longer period of time is often not feasible. The sequence had previously been optimized for the brain and translation to the abdomen required only minor adaptations (i.e., longer RF pulse duration).

Comparative literature on abdominal ^2^H relaxation times is sparse [19, 20, 37], presumably because DMI relaxation time experiments in the abdomen are even more challenging than in the brain. Simple unlocalized acquisitions, as often used in the brain for relaxation time experiments [1, 10, 26, 38], are no longer feasible since the abdomen yields a composite signal from multiple organs. Compared to volume coils, transmit/receive surface-coil arrays feature limited penetration depth and increased *B*_1_^+^ inhomogeneities, while large coil dimensions require higher reference voltages and consequently higher energy deposition (SAR). Furthermore, lower SNR requires larger voxel size, leading to increased partial-volume contamination. Additionally, relaxation measurements that involve tracers are time-sensitive because metabolite concentrations remain in a (semi)-steady-state only for a brief period.

All these effects pose challenges in abdominal relaxation time estimation. The employed surface coil provides a highly uniform *B*_1_^+^ extending at least 10 cm into the abdomen [24], which is important to ensure full inversion, refocusing or excitation, but requires high references voltages (800 V for a 0.5 ms rectangular excitation pulse). Given the low gyromagnetic ratio of deuterium, the resulting B₁⁺ distribution is expected to be largely object-independent, and phantom measurements therefore provide a realistic approximation of the in-vivo transmit field. Despite the coil’s uniform *B*₁⁺ profile, achieving full inversion and refocusing throughout the abdomen remains challenging, particularly in deeper structures such as the kidneys. Although flip-angle calibration was carefully verified in phantom measurements, residual in vivo *B*₁⁺ variation cannot be fully excluded. For this reason, *T*₁ relaxation was assessed using inversion recovery, which is considered the most robust method to measure longitudinal relaxation [39], in combination with a three-parameter fit, including an additional degree of freedom to account for incomplete inversion. For *T*₂ experiments deviations from an ideal 180° refocusing pulse or 90° excitation pulse are expected to reduce overall SNR but does not introduce a systematic bias to estimate *T*₂ relaxation times.

To maintain an acceptable scan time while achieving a sufficient longitudinal recovery, we selected a minimum *TR* approximately three times the expected HDO *T*₁ [40]. Although this *TR* potentially does not permit full relaxation, inversion recovery remains accurate under these conditions due to the intrinsic symmetry of the inversion-recovery signal (− *M*_z_ to + *M*_z_), and the three-parameter fit further mitigates the impact of incomplete recovery.

Ideally relaxation times would be measured under stable metabolite levels, and care must be taken to avoid bias from metabolite concentration changes. Reported hepatic and renal ^2^H-Glc time courses show initial rise after tracer consumption followed by a semi-steady plateau after ~ 50 min, but with high inter-subject variability [18, 20, 24, 41]. Accordingly, relaxation time experiments were started approximately 50 min post-dose where relatively stable metabolite concentrations can be assumed.

Given the expense of deuterated tracer studies, a cost-efficient protocol was set up, consisting of three scans per session (two in one organ and one in the other), alternating the organ order to avoid bias. All scans were completed within 3 h of dosing. Although organ-resolved metabolite monitoring could not be implemented in the abdomen, it was shown in simulations that our developed sequence featuring specific *k*-space ordering of the concentric ring trajectories is insensitive to metabolite concentration changes and can reliably estimate relaxation constants even under non-steady metabolite concentrations, as might be the case towards the end of the measurement. Simulations showed that, even with monotonic concentration changes of ± 35% during simulated acquisition, estimated relaxation times deviated by only 0–3% from a steady-state gold standard [27].

The original implementation of the sequence also features interleaved unlocalized FID acquisitions for metabolite concentration tracking, as used previously for brain ^2^H-Glc and ^2^H-Glx metabolite level monitoring. However, this feature could not be translated to abdominal relaxation time experiments since acquired unlocalized abdominal FIDs are dominated by a strong stomach signal and cannot be separated in liver and kidney ^2^H-Glc levels. Extraction of temporal information from the first *TI*/*TE* points was not feasible, as inversion-recovery and spin-echo readouts differ in contrast and SNR, preventing comparison of glucose signal amplitudes over time. Moreover, organ-specific glucose metabolism together with spatially varying coil sensitivity further prevents consistent interpretation of temporal changes, making meaningful assessment of ^2^H-Glc time-courses not feasible.

Generally, stomach-related contamination is a known challenge in abdominal DMI studies when the ^2^H-labeled substrates are administered orally [17, 18, 24]. In our study, this effect is already minimized as the MRSI scans do not start immediately after dosage, when the tracer is still predominantly in the stomach. Nonetheless, individual gastric emptying led to residual contaminating ^2^H-Glc signals from the stomach or smaller intestines, due to partial volume effects in some subjects. Voxels exhibiting unreasonably high glucose signal were conservatively excluded using a threshold of ^2^H-Glc/^2^H-water ratio > 1.5. 

Voxelwise mapping at natural abundance was feasible in liver for ^2^H-water (*T*₁/*T*₂). In the kidney for ^2^H-water and in both organs for ^2^H-Glc relaxation times, voxelwise SNR was insufficient to obtain reliable fits across the whole organ in the majority of the subjects, most likely as a result of the greater distance between kidney and surface coil and lower SNR of ^2^H-Glc compared to ^2^H-water. Thus, analogous to brain relaxation time data, spectral data was averaged within manually segmented liver and kidney masks before spectral fitting, which substantially increased the SNR and allowed stable fitting of organ-specific relaxation time constants of ^2^H-water and ^2^H-Glc after tracer administrations. This approach aligns well with expected physiology of the organs: resolving kidney cortex-medulla differences would require finer spatial resolution than feasible, and the liver is assumed comparatively homogeneous, making organ-averaged reporting appropriate. Low-rank spectral denoising (tensor Marchenko-Pastur PCA, tMPPCA) was additionally applied, as recently shown to be effective for very low-SNR metabolite data, further improving SNR [24, 42, 43].

^2^H-Glc *T*_1_ and *T*_2_ relaxation times were between 55–105 ms and 25–39 ms, respectively, with significant difference detected for *T*_1_ relaxation between the two organs. Inter-subject variability was lower in liver (COV = 7%) than kidney (COV = 22%). Several factors likely contributed to the higher renal variability: depth-dependent B₁⁺ that can reduce inversion efficiency in deeper renal tissue, smaller kidney mask volumes for spectral averaging that lower SNR, and more challenging *B*₀ shimming in kidney. In contrast, the liver is in closer proximity to the surface coil and exhibited roughly 50% higher SNR^HDO^ and two-fold lower CRLBs, resulting in more robust fitting. To mitigate depth-dependent *B*₁⁺ losses, the inversion-pulse reference voltage for *T*_1_ kidney measurements was increased by 25% to improve inversion efficiency. This was effective, nevertheless, it should be noted, that full inversion could not be achieved in some volunteers, presumably due to a combination of imperfect *B*_0_ shimming and shorter *T*_1_^2^H-Glc relaxation. The three-parameter inversion-recovery model also accounts for potential imperfect inversion and should therefore still provide correct and reliable *T*_1_ estimates. Additionally, ^2^H-Glc/^2^H-water ratios at the first *TI*/*TE* were used as a measure for available glucose signal for spectral fitting. Ratios were approximately twofold higher in liver than kidney yet were sufficient for fitting. Two kidney datasets (volunteers 06 and 08) showed ratios < 0.1. For volunteer 06, the low ratio may reflect faster individual glucose turnover and/or suboptimal inversion, which could explain the longer estimated *T*₁ as a result of reduced fitting stability. In volunteer 08, despite a similarly low ratio, the *T*₁ estimate remained within the expected range. Nevertheless, considering all these factors and the limited sample size, inter-organ comparisons should still be interpreted with caution.

Comparative literature on deuterium relaxation in abdominal organs remains sparse as most prior DMI studies emphasize brain relaxation [10, 26, 27, 38] or dynamic metabolic studies [2, 9–11, 16, 18, 20, 24, 44] rather than organ-specific relaxation constants. For human liver, a saturation-recovery approach with variable *TR* yielded *T*_1_ of 85 ± 8 ms for ^2^H-Glc [20], and a separate study reported human hepatic ^2^H-glucose *T*_2_^*^ of 9.6 ± 1.3 ms, estimated from the mean FWHM of the ^2^H-Glc signal using echo-planar imaging [19], both studies were performed at 7T field strength. Our reported values are consistent with the present literature values, with our ^2^H-Glc *T*_1_ values slightly shorter and as expected ^2^H-Glc *T*_2_ constants longer than reported *T*_2_^*^. Shorter longitudinal relaxation times may possibly be caused by differences in the coil setup (dual tuned vs triple tuned) or using different methods for estimating *T*_1_ (saturation-recovery vs. inversion recovery). ^2^H-Glc relaxation times for human kidney have not been reported to the best of our knowledge.

In hepatic ^2^H-DMI, ^2^H-glycogen resonates near its precursor ^2^H-Glc but is effectively invisible in vivo due to its extremely short *T*₂ (< 2 ms) [23]. Accordingly, relaxation times for glycogen were not reported and the ~ 3.9 ppm signal is attributed to glucose.

^2^H-water relaxation times were estimated without tracer administration and as echo times for scans performed after ^2^H-Glc administration were identical to the natural abundance *T*_2_ protocol allowed estimation of *T*_2_ HDO constants. A direct comparison showed no significant difference between *T*₂ values estimated at natural abundance and those obtained after glucose administration. HDO relaxation times were overall in good agreement with prior work all conducted at 7T field strength: for the human liver *T*_2_* of 13 ± 1 ms was reported [19] and longer hepatic *T*_1_ of 341 ± 14 ms using relaxation recovery [20]; kidney values fall in the same range as relaxation time values measured in rabbit kidneys (*T*_1_ = 318 ± 8 ms, *T*_2_ = 72 ± 9 ms) [37]. Significantly shorter *T*_1_ and *T*_2_ relaxation time constants were estimated in the liver compared to the kidney. As deuterium relaxation is dominated by quadrupolar rather than dipolar interactions, differences in organ iron content are unlikely to account for the observed effect. Although paramagnetic substances such as iron can accelerate transverse proton relaxation [45], early NMR studies showed that ^2^H water linewidths remain stable even in iron-loaded tissue where proton spectra are severely broadened [46]. Paramagnetic agents must reach concentrations orders of magnitude higher than those affecting protons to measurably alter ^2^H relaxation [47], and recent 7T data confirmed this insensitivity, with iron-rich regions showing elevated proton *R*₂* but unchanged deuterium *R*₂* [38]. The observed contrast between hepatic and renal ^2^H relaxation times is, therefore, more likely a result of differences in tissue composition and microstructural differences that lead to different rotational correlation times of water molecules [48]. The liver’s higher cellular density, abundant macromolecular content, and complex protein-membrane lengthen rotational correlation times, leading to shorter *T*_1_ and *T*_2_ values [49, 50]. In comparison, the kidneys feature more structured and organized water compartments within tubules and glomeruli, as well as lower overall macromolecular content, leading to longer relaxation times [51, 52]. This is also consistent with trends reported in proton relaxation literature [53, 54], since *T*_1_ and *T*_2_ share similar functional dependences for quadrupolar and dipolar relaxation mechanism [23, 48].

High-resolution renal data were acquired in one healthy volunteer after consuming D_2_O the day prior to the measurement as proof of concept, which substantially increase the SNR enabling renal mapping of *T*_1_ and *T*_2_ relaxation times with sub-milliliter isotropic resolution. Consistent with proton literature showing shorter relaxation in renal cortex compared to the medulla [54], ^2^H-water maps suggest shorter *T*_1_ and *T*_2_ constants in the outer border of the kidney. While areas of believed renal hilum displayed unexpectedly shorter *T*_1_ values, potentially due to imperfect *B*₀ shimming or partial-volume effects, *T*₂ values were longer in the expected hilum, consistent with high vascular content. However, these regional assignments remain presumptive, as no segmentation (cortex, medulla, hilum) was performed and so far, only one volunteer was measured to demonstrate feasibility, thus apparent differences should be interpreted with caution. ^1^H anatomical images were sufficient for localization, shimming and simple manual segmentation of kidney and liver masks, but reliable intra-renal or intra-hepatic tissue segmentation was not feasible and would require higher-quality and contrast ^1^H images that cannot be obtained with the current setup at 7T. Alternatively, higher-quality ^1^H images could be acquired at lower field strength (i.e., 3T) for tissue segmentation and then co-registered to 7T ^2^H-MRSI data.

Mean map-based values (after spectral fitting) were comparable with averaged values (before spectral fitting), with deviations < 10%. It should be noted that the volunteer scanned at higher resolution after D₂O dosing exhibited the longest HDO* T*₁. Because these measurements were acquired only in a single subject to demonstrate feasibility, reproducibility was not assessed. As neither D₂O administration nor increased spatial resolution is expected to affect relaxation times, this deviation most likely reflecting inter-subject variability. In principle the SNR could be increased further by consuming D_2_O over several days before the measurement, which would potentially allow to even further increase spatial resolution.

Knowledge of accurate relaxation times is essential for quantitative ^2^H-MRSI since relaxation constants are needed to correct arbitrary units into concentrations (mM) [25]. In prior abdominal DMI studies, relaxation times were either assumed to be comparable with reported tissue-specific brain values [24] or derived from FWHM [19], which is prone to systematic bias. The organ-specific values reported in this study provide a solid basis for reliable concentration estimation in future abdominal DMI studies.

## Conclusion

We successfully implemented 3D CRT based ^2^H-MRSI in the abdomen, which allowed assessing ^2^H-water and ^2^H-Glc relaxation times in the human liver and kidney without tracer administration and after oral administration of ^2^H-labeled Glc. As a proof of concept, one volunteer was measured after D₂O loading, which substantially increased voxelwise SNR and enabled sub-milliliter renal *T*₁ and *T*₂ mapping. The reported organ-specific relaxation constants are an important step in the ongoing process of clinical adoption of abdominal DMI, which can improve concentration estimation of future abdominal DMI studies to better understand the underlying metabolic model in renal and hepatic physiology and pathology.

## Supplementary Information

Below is the link to the electronic supplementary material.

Supplementary file1 (DOCX 265 KB)

## Data Availability

Data generated by postprocessing methods (i.e., metabolic maps, LCModel basis sets, Python and MATLAB script files for data analysis and plotting) are available from the corresponding author on reasonable request for research purposes only.

## Abbreviations

COV: Coefficient of variation
CRT: Concentric ring trajectory
DMI: Deuterium metabolic imaging
FID: Free induction decay
Glc: Glucose
^2^H: Deuterium
^1^H: Proton
MRSI: Magnetic resonance spectroscopic imaging
SAR: Specific absorption rate
SNR: Signal to noise ratio
tMPPCA: Tensor marchenko-pastur PCA
