# Supplementary material for: Assessment of T1 and T2 relaxation times of deuterium (2H) labeled resonances in the human liver and kidney using k-space reordered 3D concentric ring trajectory sampling at 7T
Source: MAGMA. 2026 Jan 16;39(3):499–512. doi: 10.1007/s10334-025-01320-9 (PMC13354620; doi:10.1007/s10334-025-01320-9)
Supplement: Supplementary file 1 — Supplementary file1 (DOCX 265 KB) [file 10334_2025_1320_MOESM1_ESM.docx]

# Supplementary material

| *Minimum Reporting Standards in MR Spectroscopy checklist (according to Lin et al. NMR Biomed 2021)* | |
| --- | --- |
| **1. Hardware** |  |
| *a. Field strength [T]* | 7T |
| *b. Manufacturer* | Siemens |
| *c. Model (software version if available)* | Magnetom dot Plus |
| *d. RF coils: nuclei (transmit/ receive), number of channels, type, body part* | ^2^H/^1^H dual tuned body coil array, transmit/receive, 1 channel transmit / 2 channels receive, (Stark Contrast MRI Coils Research, Germany) |
| *e. Additional hardware* | N/A |
| **2. Acquisition** |  |
| *a. Pulse sequence* | 3D FID-acquire density-weighted concentric ring trajectory (CRT) MRSI with interleaved Inversion Recovery / Hahn spin-echo acquisitions, unlocalized FID-acquire reference scans |
| *b. Volume of Interest (VOI) locations* | kidney, liver; unlocalized excitation using rectangular RF pulse with 90° flip angle |
| *c. Nominal VOI size [cm^3^, mm^3^]* | FOV 270x270x260 mm^3^ |
| *d. Repetition Time (TR), Echo Time (TE) [ms, s]* | CRT acquisition delay : 2/2.5ms (T_1_ ^LR/HR^), 0ms (T_2_)  TR = 900-1150ms  Water:   - TI: 5, 50, 450, 650, 900ms; TE: 8, 10, 15, 20,40,60ms   Glc:   - TI: 5, 15, 70, 150, 500ms; TE: 8, 10, 15, 20,40,60ms |
| *e. Total number of Excitations or acquisitions per spectrum* | Low resolution (LR): 21 circles + averaged over kidney or liver masks High resolution (HR): equidistant sampling |
| *In time series for kinetic studies* | N/A |
| *i.         Number of Averaged spectra (NA) per time-point* | N/A |
| *ii.       Averaging method (e.g. block-wise or moving average)* | N/A |
| *iii.      Total number of spectra (acquired / in time-series)* | N/A |
| *f. Additional sequence parameters (spectral width in Hz, number of spectral points, frequency offsets); If STEAM: Mixing Time TM; If MRSI: 2D or 3D, FOV in all directions, matrix size, acceleration factors* | CRT MRSI: BW: 380 Hz, 96 spectral points, LR: 18x18x17 or HR: 32x32x31 |
| *g. Water Suppression Method* | No water suppression |
| *h. Shimming Method, reference peak, and thresholds for “acceptance of shim” chosen* | Standard DESS+GRE-BREAST shim + manual adjustment, ^1^H water peak < 100 Hz, global ^2^H water peak < 70 Hz Region: both kidneys or liver |
| *i. Triggering or motion correction method (respiratory, peripheral, cardiac triggering, incl. device used and delays)* | - |
| **3. Data analysis methods and outputs** |  |
| *a. Analysis software* | LCModel 6.3-1 |
| *b. Processing steps deviating from quoted reference or product* | N/A |
| *c. Output measure (e.g. absolute concentration, institutional units, ratio)* | - |
| *d. Quantification references and assumptions, fitting model assumptions* | Simulated in NMRScope-B |
| **4. Data Quality** |  |
| *a. Reported variables (SNR, Linewidth (with reference peaks))* | SNR and linewidths reported see Table S2 |
| *b. Data exclusion criteria* | LCModel SNR >3, |
| *c. Quality measures of postprocessing Model fitting (e.g. CRLB, goodness of fit, SD of residual)* | CRLB |
| *d. Sample Spectrum* | See Figure 2, Figure 5 |

**Supplementary Table 1:** Minimum Reporting Standards for in vivo MR Spectroscopy

**
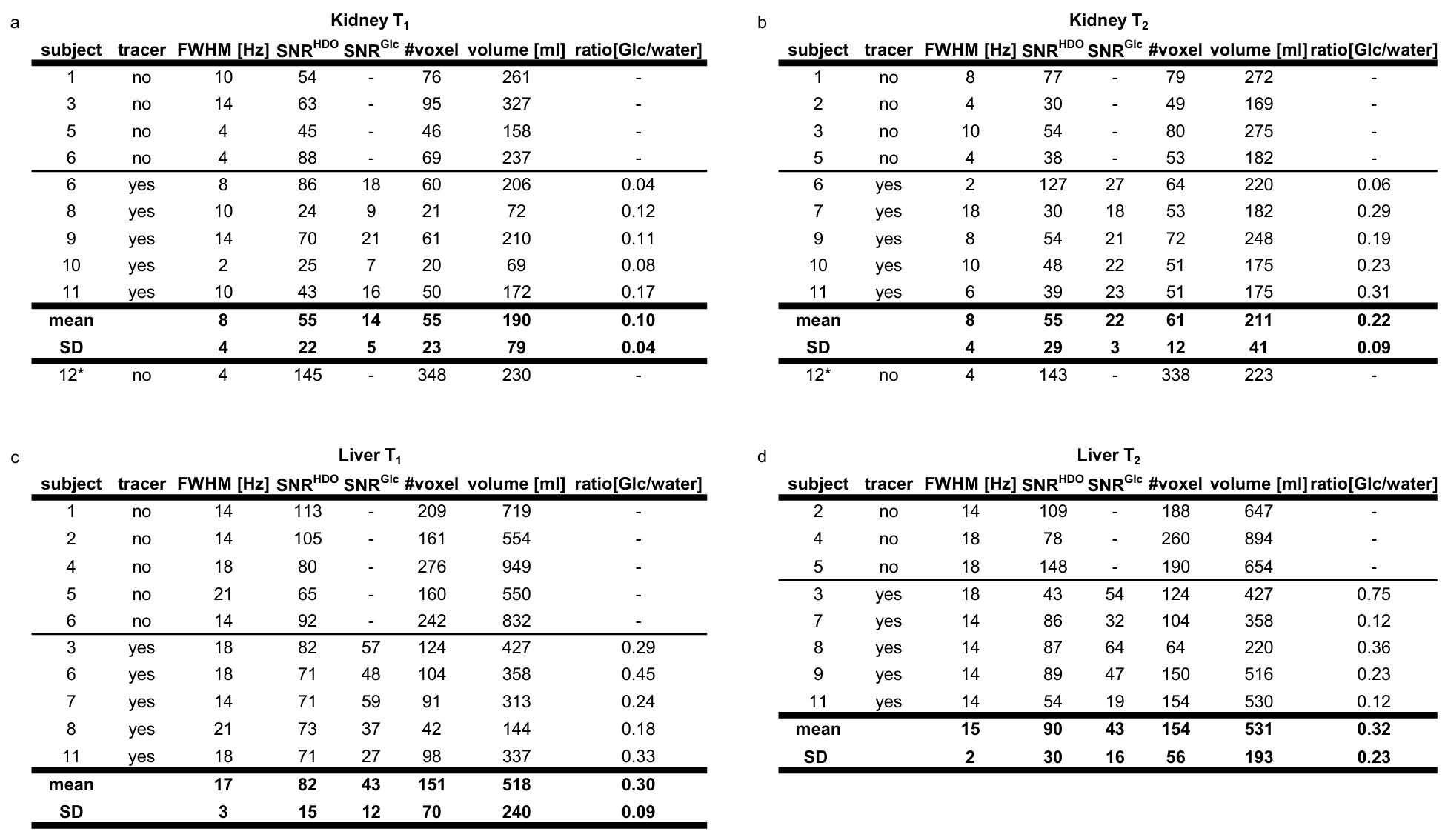
**

**Supplementary Table 2:** Overview of SNR^HDO/Glc^, FWHM (water peak) number of averaged voxels and averaged voxel volume for kidney (*T*_1_: a, *T*_2_: b) and Liver (*T*_1_: c, *T*_2_: d) for each volunteer for scans without tracer (natural abundant water) and after oral administration of ²H-Glc-tracer. One subject (12) was measured with higher spatial resolution.
